# Perinatal management of trisomy 18: a survey of obstetricians in Australia, New Zealand and the UK

**DOI:** 10.1002/pd.4249

**Published:** 2013-10-30

**Authors:** D J C Wilkinson, L de Crespigny, C Lees, J Savulescu, P Thiele, T Tran, A Watkins

**Affiliations:** 1 Robinson Institute, University of AdelaideAdelaide, Australia; 2 Oxford Uehiro Centre for Practical Ethics, University of OxfordOxford, UK; 3 Fetal Medicine, Queen Charlotte's and Chelsea Hospital, Imperial College Healthcare NHS TrustDu Cane Rd, London, W12 0HS; 4 Monash UniversityFrankston, Australia; 5 Mercy Hospital for WomenMelbourne, Australia

## Abstract

**Objective:**

The objective of this study was to explore the attitudes of obstetricians in Australia, New Zealand and the UK towards prenatally diagnosed trisomy 18 (T18).

**Method:**

Obstetricians were contacted by email and invited to participate in an anonymous electronic survey.

**Results:**

Survey responses were obtained from 1018/3717 (27%) practicing obstetricians/gynaecologists. Most (60%) had managed a case of T18 in the last 2 years. Eighty-five per cent believed that T18 was a ‘lethal malformation’, although 38% expected at least half of liveborn infants to survive for more than 1 week. Twenty-one per cent indicated that a vegetative existence was the best developmental outcome for surviving children. In a case of antenatally diagnosed T18, 95% of obstetricians would provide a mother with the option of termination. If requested, 99% would provide maternal-focused obstetric care (aimed at maternal wellbeing rather than fetal survival), whereas 80% would provide fetal-oriented obstetric care (to maximise fetal survival). Twenty-eight per cent would never discuss the option of caesarean; 21% would always discuss this option. Management options, attitudes and knowledge of T18 were associated with location, practice type, gender and religion of obstetricians.

**Conclusion:**

There is variability in obstetricians' attitudes towards T18, with significant implications for management of affected pregnancies. © 2013 The Authors. *Prenatal Diagnosis* published by John Wiley & Sons, Ltd.

## INTRODUCTION

Trisomy 18 (T18) is one of the commonest major chromosomal abnormalities,^1^ affecting about 1/4000 live births.^2^ There appears to be a rising incidence due to increasing maternal age as well as the influence of early aneuploidy screening (with recognition of T18 in some fetuses who miscarry).^3^ However, birth prevalence remains steady because of termination of pregnancy following antenatal diagnosis.^3^,^4^

In the last decade, there has been a shift in attitude of paediatricians towards T18.^5^,^6^ This follows publication of evidence that despite a high rate of fetal or neonatal death, a small proportion of affected infants are able to survive infancy. This has been coupled with increasing parental requests for active treatment.^5^,^6^ Long-term survival is associated with severe to profound developmental disabilities.^7^,^8^ The majority of fetuses and infants with T18 have cardiac defects, although these range in severity.^9^ Cardiac surgery is offered in some parts of the world^10^,^11^ but not in others. This raises challenging ethical questions about management of affected fetuses and infants.^8^,^12^

A number of guidelines have been written about obstetric management following diagnosis of severe malformations such as T18. There are strong recommendations that counselling about termination should be non-directive^13^,^14^ and should avoid the term ‘lethal’.^15^ If a pregnancy continues, it has been recommended that women are offered the option of maternal-focused obstetric management (aimed at maternal wellbeing rather than fetal survival).^16^,^17^ However, ethicists and commentators have argued that it is also appropriate for women to be given the option of fetal-oriented care (aimed at maximising fetal survival), for example including fetal monitoring during labour and/or caesarean section (CS).^17^,^18^

Recent accounts by women who have experienced antenatal diagnosis of T18 have described a relative lack of support once they expressed a desire not to terminate their pregnancy.^19^–^21^ Parent narratives commonly describe a desire for live birth if possible, with varying degrees of postnatal intervention. It is not known how obstetricians respond to requests for fetal-oriented obstetric care.

A recent paper examined the attitudes of US obstetricians towards management options for severe fetal malformations (for example T18).^22^ There are no available data on the knowledge or attitudes of obstetricians in Australia, New Zealand or the UK. We sought to explore the attitudes of doctors working in these countries to prenatal, intrapartum and postpartum care when a pregnancy is affected by T18.

## METHODS

Fellows of the Royal Australian and New Zealand College of Obstetrics and Gynaecology (RANZCOG) and fellows and members of the Royal College of Obstetrics and Gynaecology (RCOG) in the UK were contacted by email and invited to participate in an anonymous electronic survey. Non-respondents were sent two reminder emails.

The survey had been developed by the study authors after a review of the medical literature. It was validated and modified following pilot surveys with practising obstetricians. The survey included basic demographic questions (gender, practice type, years of experience and religion) and questions about experience, knowledge and attitudes towards T18. Questions about religion were based on the British Social Attitudes survey.^23^ At the request of RANZCOG, Australian and New Zealand (A/NZ) participants were not asked about their religious beliefs. For attitudes towards T18, respondents were asked their level of agreement on six-point Likert scales. Participants were presented with a hypothetical case of a woman whose pregnancy was diagnosed with T18 at 13 weeks and were asked about treatment options they would discuss or offer following diagnosis. In the event that the woman's pregnancy continued, participants were asked about further management provided during pregnancy and around the time of delivery. Participants were specifically asked how they would respond to a request by the patient for either maternally focused or fetally oriented obstetric care using questions derived from a recent US survey.^22^

Statistical analysis was carried out using sas software, version 9.3 (SAS Institute Inc, Cary, NC, USA). The findings were descriptively presented as frequency (%) for discrete variables and mean (standard deviation) or median (inter-quartile range), where appropriate, for continuous variables. The association between baseline characteristics and outcomes of interest were explored using the Pearson chi-square test for independence or Fisher's exact test, where appropriate, for discrete variables, and Student's *T* test or Mann–Whitney *U* test, where appropriate, for continuous variables. We performed logistic regression models to determine the independent effect of baseline characteristics including location (UK vs Australia), gender (female vs male), years of practising as a specialist, main practice (obstetrics, ultrasound or maternal–fetal medicine vs other) and main practice location (tertiary vs other) on the following outcomes: expectation of survival or development for T18, discussion or provision of termination of pregnancy, and maternal or fetal-oriented obstetric management. (Religion was excluded from the multivariate analysis as this information was only available for UK respondents.) For Likert scale responses, ‘strongly agree’ and ‘moderately agree’ were grouped together, as were ‘strongly disagree’ and ‘moderately disagree’. A *p*-value of 0.05 or less was considered to indicate statistical significance.

## RESULTS

### Sample

We wrote to 3820 obstetricians/gynaecologists by email. There were 1121 replies, of whom 103 indicated that they were not usually involved in the care of pregnant women and were excluded. We analysed 1018 responses from practising obstetricians, including 436 responses from A/NZ (47% of those contacted) and 582 from UK (20% of those contacted). Table 1 indicates baseline demographic information for respondents in A/NZ and UK. Because UK senior trainees are members of the RCOG, whereas RANZCOG Fellowship is only awarded on completion of training, there were more trainees in the UK cohort, there being none in the A/NZ cohort.

**Table 1:** Baseline characteristics of participants by location

Characteristics	Overall, *n* (%)	Australia, *n* (%)	UK, *n* (%)	*p*-value^a^
Gender (*n* = 920)^b^				
Male	440 (47.8)	236 (58.0)	204 (39.8)	<0.0001
Female	480 (52.2)	171 (42.0)	309 (60.2)	
Years practising as a specialist (*n* = 932)^b^				
Still in training	52 (5.6)	0	52 (10.0)	<0.0001
<5 years	143 (15.3)	60 (14.6)	83 (16.0)	
5–15 years	330 (35.4)	141 (34.2)	189 (36.4)	
15–25 years	254 (27.3)	107 (26.0)	147 (28.3)	
>25 years	153 (16.4)	104 (25.2)	49 (9.4)	
Main practice (*n* = 1015)^b^				
Obstetrics	619 (61.0)	294 (67.7)	325 (55.9)	<0.0001
Gynaecology	214 (21.0)	75 (17.3)	139 (23.9)	
Maternal–fetal medicine	140 (13.8)	43 (9.9)	97 (16.7)	
Ultrasound/prenatal testing	29 (2.9)	19 (4.4)	10 (1.7)	
Others	13 (1.3)	3 (0.7)	10 (1.7)	
Main practice location (*n* = 1015)^b^				
Tertiary hospital	336 (33.1)	144 (33.2)	192 (33.1)	<0.0001
District general/regional public hospital	509 (50.1)	134 (30.9)	375 (64.5)	
Private hospital	73 (7.2)	69 (15.9)	4 (0.7)	
Private practice	89 (8.8)	84 (19.4)	5 (0.9)	
Other	8 (0.8)	3 (0.7)	5 (0.9)	
Religion^c^ (*n* = 514)^b^				
Christian			222 (43.2)	N/A
Hindu			108 (21.0)	
Muslim			14 (2.7)	
Other			98 (19.1)	
None				
Strength of religion^c^ (*n* = 515)^b^				
Religious			275 (53.4)	N/A
Non-religious			101 (19.6)	
Neither religious nor non-religious			139 (27.0)	

a Chi-squared test or Fisher's exact test where appropriate, comparing responses by location.

b *n* refers to the number of participants responding to that question (participants did not always answer every question).

c Questions about religion were only asked of participants from the UK.

### Personal experience

Sixty per cent of respondents indicated that they had been involved in managing a case of antenatally diagnosed T18 in the last 2 years, and 8% had never managed a case of T18; 37% of respondents had managed at least five cases. Most respondents (70%) indicated a high rate (≥95%) of termination of pregnancy in cases with which they had been personally involved.

### Attitudes and expectations

A high proportion (85%) of respondents moderately or strongly agreed with a statement that T18 was a ‘lethal malformation’ (Table 2), although 38% indicated that from their experience and understanding at least half of liveborn infants would survive for more than 1 week with treatment. Seventy-one per cent of respondents expected 5-20% of infants to survive for more than 1 year, whereas 78% indicated that profound disability or a vegetative existence was the best developmental outcome for surviving children.

**Table 2:** Attitudes towards T18, and expectations of outcome

Attitudes towards T18 (*n* = 962)^a^	Strongly/moderately disagree, *n* (%)	Strongly/moderately agree, *n* (%)
T18 is a lethal abnormality	72 (7.5)	813 (84.5)
A fetus with T18 should be treated no differently from any other fetus	721 (75.0)	110 (11.4)
Active treatment of a fetus or newborn with T18 is futile	163 (16.9)	601 (62.5)
T18 is compatible with the child having a meaningful life	759 (78.9)	79 (8.2)
T18 is incompatible with life	274 (28.5)	521 (54.2)
Infants with T18 should not be resuscitated at birth (*n* = 948)^a^	269 (28.4)	421 (44.4)
		
Knowledge/expectations about outcome for fetuses with T18 (*n* = 936)^a^	Expected outcome (proportion of fetuses or newborns that respondent would expect to achieve specified outcome)	Proportion of respondents, *n* (%)
Survival to term if not terminated	None	8 (0.9)
	**5–20%**^b^	**485 (51.8)**
	50%	283 (30.2)
	≥75%	160 (17.1)
Survival for more than 1 week if liveborn and if treatment is provided	None	22 (2.3)
	5–20%	563 (60.1)
	**50%**^b^	**241 (25.8)**
	≥75%	110 (11.8)
Survival for more than 1 year if liveborn and if treatment is provided	None	219 (23.4)
	**5–20%**^b^	**666 (71.2)**
	50%	34 (3.6)
	≥75%	17 (1.8)
Best developmental outcome if survives for some time (non-mosaic)	Mild intellectual disability	34 (3.6)
	Severe disability	170 (18.2)
	**Profound disability**^b^	**540 (57.7)**
	Vegetative existence	192 (20.5)

a *n* refers to the total number of respondents who answered an individual question (participants did not always answer every question).

b Estimate of outcome that appears most consistent with population-based figures – see discussion.

### Management

Ninety-two per cent of participants indicated that they would always discuss or offer termination following antenatal diagnosis of T18 (Table 3). Twenty-one per cent (201) of respondents indicated that they had an ethical or moral objection to termination; 84% (174) of those with an objection would refer to another obstetrician who would offer termination.

**Table 3:** Treatment options for T18 discussed or offered following antenatal diagnosis

Options/responses	Overall, *n* (%)	A/NZ, *n* (%)	UK, *n* (%)	*p*-value^a^
Main management options (*n* = 962)^b^				
Termination of pregnancy				
Always/sometimes	912 (94.8)^c^	408 (96.9)	504 (93.2)	0.009
Only if asked/never	50 (5.2)	13 (3.1)	37 (6.8)	
Paediatric consultation early in pregnancy				
Always/sometimes	651 (67.7)	251 (59.6)	400 (73.9)	<0.0001
Only if asked/never	311 (32.3)	170 (40.4)	141 (26.1)	
Further management options if pregnancy continues (*n* = 953)				
If the pregnancy continues, and the woman asks you *not to* intervene on the fetus' behalf to maximise the chance of a live birth (fetal monitoring/CS), would you comply? (maternal-focused obstetric care)	939 (98.5)	410(98.1)	529 (98.9)	0.31
If yes, how would you counsel the patient?				
Encourage	260 (27.8)	122 (29.8)	138 (26.2)	0.002
Support, but not encourage	390 (41.7)	147 (35.9)	243 (46.2)	
Neither encourage nor discourage	255 (27.2)	120 (29.3)	135 (25.7)	
Discourage	31 (3.3)	21 (5.1)	10 (1.9)	
If the pregnancy continues, and the woman asks you *to* intervene on the fetus' behalf to maximise the chance of a live birth (fetal monitoring, CS), would you comply? (fetal-oriented obstetric care)	755 (79.5)	335 (80.1)	420 (79.0)	0.65
If yes, how would you counsel the patient?				
Encourage	22 (2.9)	13 (3.89)	9 (2.1)	0.34
Support, not encourage	269 (35.6)	116 (34.6)	153 (36.4)	
Neither encourage nor discourage	182 (24.1)	75 (22.4)	107 (25.5)	
Discourage	282 (37.4)	131 (39.1)	151 (36.0)	
Palliative care-based perinatal management				
Always/sometimes	826 (86.7)	344 (82.3)	482 (90.1)	<0.0001
Only if asked/never	127 (13.3)	74 (17.7)	53 (9.9)	
Fetal monitoring during labour				
Always/sometimes	363 (38.1)	124 (29.7)	239 (44.7)	<0.0001
Only if asked/never	590 (61.9)	294 (70.3)	296 (55.3)	
CS for fetal distress				
Always/sometimes	309 (32.4)	115 (27.5)	194 (36.3)	0.004
Only if asked/never	644 (67.6)	303 (72.5)	341 (63.7)	
Paediatrician present at birth				
Routinely	618 (65.2)	250 (60.0)	368 (69.3)	0.009
If requested	311 (32.8)	156 (37.4)	155 (29.2)	
Never	19 (2.0)	11 (2.6)	8 (1.5)	

a Chi-squared test comparing A/NZ responses with UK responses.

b *n* refers to the total number of respondents who answered an individual question (participants did not always answer every question).

c 91.5% of respondents indicated that they would ‘always’ discuss or offer termination, and 3.3% would discuss termination ‘sometimes’.

In the event of a continuing pregnancy, 99% of respondents would comply with a request for maternal-focused obstetric care, with 28% encouraging this request. Eighty per cent of respondents would comply with a request for fetal-oriented obstetric care in the same situation, although 37% would discourage this approach. Twenty-three per cent of respondents would never discuss or offer fetal monitoring in labour, whereas 24% would always offer this option. Twenty-eight per cent would never offer CS in the event of fetal distress, whereas 21% would always discuss this option.

### Influence of location

There were significant differences between respondents from the UK and A/NZ in baseline characteristics (Table 1). A higher proportion of UK respondents were female, in training or worked in district/general hospitals. Australian respondents were more likely to have >25 years of experience, to work predominantly in private practice and in obstetrics. Management of pregnancies following diagnosis of T18 also differed by location (Table 3 and Table S1). A higher proportion of A/NZ respondents would always or sometimes offer termination and pastoral care in the event of a continuing pregnancy. Respondents from the UK were more likely to discuss or offer paediatric consultation, palliative care, multidisciplinary team involvement, fetal monitoring and CS. Similar proportions of respondents would comply with requests for either maternal-oriented or fetal-oriented obstetric care. Respondents from the UK had more recent experience of T18 and reported a lower proportion of pregnancies followed by termination. Attitudes towards T18, and expectations of outcome were similar (data not shown).

### Gender

There was a higher proportion of female respondents from the UK (60% vs 42%, *p* < 0.0001). Women were more likely to have 5–15 years of practise as a specialist (44% vs 26%, *p* < 0.0001); they had managed similar numbers of cases recently, but a smaller proportion of female respondents indicated that all past cases had been followed by termination (35% vs 47%, *p* = 0.002). Female respondents were more likely to comply with a request for maternal-focused obstetric care (100% vs 97%, *p* = 0.004), but a similar proportion would comply with a request for fetal-oriented care. A similar proportion of male and female respondents were directive in counselling, actively encouraging or discouraging management options. A higher proportion of female obstetricians would discuss or offer paediatric consultation (72% vs 63%), palliative care (92% vs 81%), multidisciplinary team input (83% vs 73%), clinical ethics consultation (49% vs 38%) and pastoral care (67% vs 53%, *p* < 0.0001). A smaller proportion of female respondents expected that the majority of liveborn infants with T18 would survive for more than 12 months (3% vs 8%, *p* = 0.002).

### Religion

Amongst UK respondents, the strength of religious views appeared to influence management options. Fifty-three per cent (275) of respondents indicated that they were somewhat, very or extremely religious, whereas 20% (101) were somewhat, very or extremely non-religious, and 27% (139) were neither religious nor non-religious (neutral). Those who described themselves as religious were more likely to express an ethical or moral objection to termination (32%) than those who were non-religious (3%) or neutral (12%, *p* < 0.0001). Eighty-four per cent of religious respondents would always discuss termination following antenatal diagnosis of T18 compared with 97% of non-religious and 89% of those who were neutral (*p* = 0.007). Eight per cent of religious or neutral respondents would only discuss termination if asked or would never discuss it compared with 1% of non-religious respondents (Table S2). Of those respondents who expressed an objection to termination, 89% of religious respondents would refer to another practitioner for termination compared with 100% of non-religious respondents (*p* = NS).

Specific religious denominations were associated with attitudes to termination. Those who would not offer termination (compared with those who would discuss/offer termination) were more likely to indicate that their religious affiliation was Muslim (44% vs 12%) and less likely to be Christian (33% vs 43%) or no religion (6% vs 20%, *p* < 0.0001).

Non-religious respondents were more likely to strongly or moderately agree that infants with T18 should not be resuscitated at birth (57%) than religious (32%) or neutral (38%) respondents (*p* < 0.0001). Religious respondents were more likely to indicate that fetuses with T18 should be treated no differently from any other fetus and more likely to discuss or offer clinical ethics consultation. They had similar expectations about outcome for fetuses and infants with T18 to non-religious respondents (data not shown). The specific religion of respondents was not associated with provision of either maternal-focused or fetal-oriented obstetric care.

### Expectations of outcome

Respondents' views about the outcome for fetuses and infants with T18 were associated with management options that would be provided on request. A higher proportion of respondents who would not comply with a request for maternal-focused obstetric care (compared with those who would comply) expected a high chance (≥50%) of long-term survival for infants (29% vs 5%, *p* < 0.0001). Respondents who would comply with a request for obstetric care to maximise fetal survival (compared with those who would not comply with such a request) were more likely to believe that at least half of infants with T18 survive for more than 1 week (40% vs 26%) or 1 year (6% vs 2%) and that mild intellectual disability (4% vs 1%) or severe disability (21% vs 9%) were the best developmental outcomes (all *p* < 0.0001).

### Factors influencing management options and expectations for T18 (multivariate analysis)

Multivariate analysis indicated that physician location and type of practice influenced whether respondents would discuss/offer termination. UK respondents were less likely to discuss/offer termination than A/NZ respondents (OR 0.43, *p* = 0.02), whereas those working in tertiary centres were more likely to discuss/offer termination than those working in other settings (OR 3.5, *p* = 0.005). Female respondents were more likely to comply with a request for maternal-focused obstetric management (OR 7.8, *p* = 0.009). Respondents with longer experience in practice were more likely to indicate that a high proportion of fetuses with T18 survive to term (OR 1.8, *p* < 0.001), for more than 1 week (OR 1.4, *p* = 0.02) and for more than 1 year (OR 2.7, *p* = 0.003); whereas those with fetus-related specialities were less likely to indicate that 50% or more of fetuses with T18 survive for more than 1 year (OR 0.5, *p* = 0.02). There was no influence of experience or other variables on expected developmental outcome.

## DISCUSSION

This survey provides an insight into the views, knowledge and experience of UK and A/NZ obstetricians about management and counselling of antenatally diagnosed T18.

Our study provides valuable information about obstetricians' understanding of outcome for T18. Just over half believed that T18 was ‘incompatible with life’; more indicated that T18 was a ‘lethal’ anomaly. However, in an apparent contradiction, most anticipated the possibility of long-term survival if treatment was provided. The meaning of the term ‘lethal’ is potentially unclear and is a source of confusion in the minds of women.^15^,^20^,^24^ We have argued elsewhere that it should be avoided.^15^

Most respondents in the survey indicated correctly that 5–20% of fetuses would survive to full term if not terminated. Recent studies have documented a high (87–88%) rate of intrauterine death for fetuses with T18.^1^,^25^ In contrast, where the diagnosis is made after 20 weeks, a higher proportion (˜50%) of fetuses survive to live birth; this may reflect the influence of placental mosaicism,^26^ as well as the fact that fetuses with more severe structural abnormalities are likely to have been diagnosed earlier.^25^,^27^,^28^

Obstetricians' understanding of postnatal survival was commonly incorrect. Most expected that less than a quarter of liveborn T18 infants would survive for a week or more. In population-based studies, the median survival for T18 is approximately 6–10 days, with 43% surviving for more than 1 week.^29^,^30^ Approximately 5–10% of infants survive for more than 1 year.^8^,^29^ Furthermore, there are reports of 1-year survival of 25% or more in cohorts of infants treated more actively including cardiac surgery,^10^ raising the possibility that lower survival figures may represent a ‘self-fulfilling prophecy’.^31^

There were variable views amongst obstetricians about the best developmental outcome for surviving children with T18. Twenty per cent expected a ‘vegetative’ existence, whereas 4% anticipated only mild disability, views that do appear not to be supported by the literature. Older children with full (non-mosaic) T18 have a developmental age of about 6–8 months overall but are able to interact with their environment and with their family.^7^ They can use a few words or signs and play independently. Families of these children have expressed distress at overly negative information given in counselling about long-term outcome.^7^,^24^ There may be a role for further education to improve obstetricians' knowledge of potential outcome in T18.

Ninety-two per cent of surveyed obstetricians would always discuss termination after first trimester diagnosis of T18. This compares with more than 99% of US obstetricians who would discuss termination in a similar setting.^22^ In a separate question, we asked whether respondents had an ethical or moral objection to termination. Just over 20% of respondents reported such an objection; however, many of these appeared to distinguish between different reasons for termination. Seventy-eight per cent of those with an objection would always discuss termination following first trimester diagnosis of T18.

Only nine respondents (<1%) indicated that they would never discuss termination; seven indicated that although they had an ethical objection to termination, they would refer a woman to another obstetrician for this option. Twenty-nine respondents (3%) indicated that they both objected to termination and would not refer to another practitioner. Refusal to refer in the setting of a conscientious objection conflicts with professional codes of ethics^32^,^33^ and is potentially unlawful in England^34^,^35^ and in one jurisdiction in Australia.^36^ We did not ask respondents to identify their location and thus were not able to determine the effect of this on practice. However, a small number noted in free-text responses that they worked in Northern Ireland, where access to termination has been extremely limited and referral would not be legally required.^37^

Perhaps unsurprisingly, religious affiliation of obstetricians was associated with attitudes towards termination. Those who described themselves as religious were less likely to always or usually discuss termination with women following a diagnosis of T18. A recent paper from the USA found an influence of obstetrician religion on views about directiveness of counselling: Those who were non-religious and those with theologically pluralistic views were more likely to refrain from directive counselling.^38^ Our survey found the opposite: Non-religious obstetricians had a higher proportion of directive responses, being more inclined (than ‘neutral’ or ‘religious’) to actively encourage maternal-focused obstetric care and more likely to discourage fetal-oriented care. The explanation for these different findings is not immediately clear. It may relate to population differences (USA vs UK). Alternatively, it may relate to the type of case presented.

Following a diagnosis of T18, almost all respondents would comply with a request for obstetric care focused on maternal rather than fetal wellbeing, and 28% would actively encourage this approach. A majority of obstetricians would also comply with a woman's request for obstetric care aimed at fetal survival, although 20% would not comply with this request, and more than 1/3 would actively discourage this option. These results are very similar to those reported in a recent survey of US obstetricians, where >99% would provide maternal-focused obstetric care on request for a malformation such as T18, and 82% would provide fetal-oriented obstetric care if requested.^22^ In that survey, as in ours, only 1/4 of obstetricians indicated that they would be non-directive, neither encouraging nor discouraging maternal-oriented or fetal-oriented care.

We found that obstetricians were divided about specific interventions such as fetal monitoring and CS. It is interesting that roughly four in five respondents, independent of their country of practice, would potentially offer fetal monitoring, CS and the presence of a paediatrician at delivery in the context of a pregnancy affected by T18. This is in contrast to the traditional view that it is not appropriate to monitor or intervene where a baby has a severely life-limiting prognosis.^39^,^40^ It may, however, reflect current practice in some countries. In a study of live births in Texas from 1999 to 2005, 41% of infants with a prenatal diagnosis of T18 were delivered by CS.^41^ In an Internet-based survey of families of children with T18, half had been offered the option of CS.^42^

It would be interesting to ask practitioners why they sought to influence women's choice of obstetric management, in particular, avoiding fetally oriented management. One potential reason is harm to the woman. Obstetricians might be concerned about imposing medical risks on the woman (for example from CS) for relatively little benefit to the fetus. However, given the significant value that some women place on spending some time with a living child, this is a risk that some women would be more than willing to take. A separate concern might be that continuing a pregnancy or fetally oriented management would harm the fetus. Yet even if such infants survive for only a short period, or are profoundly impaired, it is not clear that they are thereby harmed.^43^ Finally, there might be concern about the long-term resource implications of care for a child with T18.^44^ But although this argument might justify limiting intensive care or cardiac surgery for long-term survivors (although this is contested^8^,^12^), the costs of future care are not usually thought to be sufficient grounds to justify limiting or influencing choices during pregnancy. Such considerations would apply to a much broader group of fetuses and would be arguably a form of passive eugenics.^45^

Limitations of the study include the low response rate (particularly from UK respondents), which may affect generalisability. However, our overall results are consistent with average response rates for electronic surveys in a recent meta-analysis (34%).^46^ Although we were not able to find data on type of practice and location of practice in the wider UK/A/NZ obstetrician population, the sex and duration of practice of respondents were similar to the sex/age distribution in the wider population of obstetricians in A/NZ and UK,^47^,^48^ reducing the likelihood of respondent bias (Table S3).^49^

In summary, there is significant variability in UK and A/NZ obstetricians' knowledge of, attitudes towards and management of prenatally diagnosed T18. One particularly important finding is that directive counselling is still relatively common and that some women may not be offered a full range of options for management during pregnancy or at delivery. This suggests a need for further debate within the professional community and further practitioner education in order to provide consistent and appropriate care for this uncommon but serious condition.

### Authorship

D. W. conceived of the study in conjunction with L. d. C., P. T. and A. W. D. W. designed and ran the survey, and completed the first draft of this work. P. T., A. W., L. d. C., J. S. and C. L. contributed to analysis and edited the article. T. T. provided statistical expertise, contributed to the analysis and edited the article. All authors approved of the final version of the paper.

### Ethics approval

The study was approved by the Social Sciences and Humanities Inter-divisional research ethics committee at the University of Oxford (SSD/CUREC1A/11-112), June 2011.

**WHAT'S ALREADY KNOWN ABOUT THIS TOPIC?**

- Many women choose to terminate after a prenatal diagnosis of trisomy 18, although some continue their pregnancy.
- Professional and ethical guidelines indicate that obstetricians should be non-directive in counselling and that fetal-oriented management may be appropriate.

**WHAT DOES THIS STUDY ADD?**

- Obstetricians in the UK, Australia and New Zealand vary in their management of prenatally diagnosed trisomy 18.
- Counselling is frequently directive.
- Perinatal management of trisomy 18 may be influenced by practitioner values.
