# Supplementary material for: Perinatal management of trisomy 18: a survey of obstetricians in Australia, New Zealand and the UK
Source: Prenat Diagn. 2013 Oct 30;34(1):42–9. doi: 10.1002/pd.4249 (PMC3963474; doi:10.1002/pd.4249)
Supplement: Supplementary file 1 [file pd0034-0042-sd1.doc]

TABLE S1

Additional treatment options for T18 discussed or offered following antenatal diagnosis

| **Options/Responses (n=962)** | **Overall**  **n(%)** | **A/NZ**  **n(%)** | **UK**  **n(%)** | **P-value**** |
| --- | --- | --- | --- | --- |
| Genetic consultation | |  |  |  |
| Always/Sometimes  Only if asked/Never | 778 (80.9)  184 (19.1) | 347 (82.4)  74 (17.6) | 431 (79.7)  110 (20.3) | 0.28 |
| Multidisciplinary team eg including paediatrician, geneticist, maternal-fetal medicine | |  |  |  |
| Always/Sometimes  Only if asked/Never | 747 (77.7)  215 (22.3) | 297 (70.6)  124 (29.4) | 450 (83.2)  91 (16.8) | <0.0001 |
| Delivery in tertiary centre* | |  |  |  |
| Always/Sometimes  Only if asked/Never | 572 (60.0)  381 (40.0) | 257 (61.5)  161 (38.5) | 315 (58.9)  220 (41.1) | 0.42 |
| Referral to paediatricians* | |  |  |  |
| Always/Sometimes  Only if asked/Never | 846 (89.0)  107 (11.0) | 345 (82.5)  73 (17.5) | 501 (93.6)  34 (6.4) | <0.0001 |
| Clinical ethics consultation* | |  |  |  |
| Always/Sometimes  Only if asked/Never | 418 (44.0)  535 (56.0) | 161 (38.5)  257 (61.5) | 257 (48.0)  278 (52.0) | 0.003 |
| Pastoral care* | |  |  |  |
| Always/Sometimes  Only if asked/Never | 577 (60.6)  376 (39.4) | 288 (68.9)  130 (31.1) | 289 (54.0)  246 (46.0) | <0.0001 |

*n=953 respondents

** chi-squared test comparing A/NZ responses with UK

TABLE S2

Influence of religion on experience, attitudes and management of prenatally diagnosed T18

| **Characteristics** |  | | **Religious**  **n(%)** | **Non-religious**  **n(%)** | **Neither**  **n(%)** | **P-value*** |
| --- | --- | --- | --- | --- | --- | --- |
| **Personal experience of T18** |  | |  |  |  |  |
| Number of cases involved in managing |  | |  |  |  |  |
| None  1-5  5-15  15-30  >30 |  | | 25 (9.1)  166 (60.4)  57 (20.7)  18 (6.5)  9 (3.3) | 4 (4.0)  44 (43.5)  23 (22.8)  17 (16.8)  13 (12.9) | 9 (6.5)  77 (55.4)  35 (35.1)  14 (10.1)  4 (2.9) | <0.0001 |
| Most recent case |  | |  |  |  |  |
| This year  Last year  Last 5 years  Last 10 years  >10 years  Never |  | | 97 (35.3)  74 (26.9)  74 (26.9)  9 (3.3)  4 (1.4)  17 (6.2) | 42 (41.5)  28 (27.7)  22 (21.8)  5 (5.0)  1 (1.0)  3 (3.0) | 47 (33.8)  54 (38.8)  27 (19.4)  4 (2.9)  1 (0.7)  6 (4.4) | 0.35 |
| Proportion elected for termination | | | | | | |
| None  25%  50%  75%  95%  100% |  | | 31 (11.3)  12 (4.4)  24 (8.7)  32 (11.6)  75 (27.3)  101 (36.7) | 4 (4.0)  4 (4.0)  7 (6.9)  15 (14.8)  43 (42.6)  28 (27.7) | 15 (10.8)  6 (4.3)  6 (4.3)  19 (13.7)  45 (32.4)  48 (34.5) | 0.14 |
| **Attitudes towards T18** |  | |  |  |  |  |
| T18 is a lethal abnormality |  | |  |  |  |  |
| Strongly/Moderately disagree  Strongly/Moderately agree  Mildly disagree/agree |  | | 26 (9.5)  229 (83.3)  20 (20.2) | 6 (5.9)  88 (87.2)  7 (6.9) | 11 (7.9)  116 (83.5)  12 (8.6) | 0.81 |
| A fetus with T18 should be treated no differently from any other fetus |  | |  |  |  |  |
| Strongly/Moderately disagree  Strongly/Moderately agree  Mildly disagree/agree |  | | 188(68.4)  47 (17.1)  40 (14.5) | 81 (80.2)  4 (4.0)  16 (15.8) | 99 (71.2)  21 (15.1)  19 (13.7) | 0.03 |
| Active treatment of a fetus or newborn with T18 is futile |  | |  |  |  |  |
| Strongly/Moderately disagree  Strongly/Moderately agree  Mildly disagree/agree |  | | 58 (21.1)  158 (57.5)  59 (21.4) | 17 (16.8)  68 (67.3)  16 (15.9) | 23 (16.5)  82 (59.0)  34 (24.5) | 0.33 |
| T18 is compatible with the child having a meaningful life | |  | |  |  |  |
| Strongly/Moderately disagree  Strongly/Moderately agree  Mildly disagree/agree |  | | 216 (78.5)  23 (8.4)  36 (13.1) | 74 (73.3)  11 (10.9)  16 (15.8) | 98 (70.5)  15 (10.8)  26 (18.7) | 0.46 |
| T18 is incompatible with life | |  | |  |  |  |
| Strongly/Moderately disagree  Strongly/Moderately agree  Mildly disagree/agree |  | | 76 (27.6)  149 (54.2)  50 (18.2) | 26 (25.7)  53 (52.5)  22 (21.8) | 36 (25.9)  75 (54.0)  28 (20.1) | 0.95 |
| T18 newborns should not receive resuscitation at birth | | | |  |  |  |
| Strongly/Moderately disagree  Strongly/Moderately agree  Mildly disagree/agree |  | | 115 (41.8)  89 (32.4)  71 (25.8) | 19 (18.8)  58 (57.4)  24 (23.8) | 41 (29.5)  53 (38.1)  45 (32.4) | <0.0001 |
| **Management options that would be discussed or offered** |  | |  |  |  |  |
| Termination of pregnancy |  | |  |  |  |  |
| Always/Sometimes  Only if asked/Never |  | | 252(91.6)  23 (8.4) | 100 (99.0)  1 (1.0) | 127 (91.4)  12 (8.6) | 0.03 |
| Obstetric management primarily for maternal health | |  | |  |  |  |
| Always/Sometimes  Only if asked/Never |  | | 242(88.0)  33 (12.0) | 90 (89.1)  11 (10.9) | 125 (89.9)  14 (10.1) | 0.84 |
| Obstetric management to maximize fetal survival | |  | |  |  |  |
| Always/Sometimes  Only if asked/Never |  | | 81(29.5)  194 (70.5) | 20 (19.8)  81 (80.2) | 34 (24.5)  105 (75.5) | 0.15 |
| Paediatric consultation early in pregnancy | |  | |  |  |  |
| Always/Sometimes  Only if asked/Never |  | | 205 (74.5)  70 (25.5) | 73 (72.3)  28 (27.7) | 104 (74.8)  35 (25.2) | 0.89 |
| Genetic consultation | |  | |  |  |  |
| Always/Sometimes  Only if asked/Never |  | | 222 (80.7)  53 (19.3) | 72 (71.3)  29 (28.7) | 112 (80.6)  27 (19.4) | 0.12 |
| Multidisciplinary team | |  | |  |  |  |
| Always/Sometimes  Only if asked/Never |  | | 236 (85.8)  39 (14.2) | 74 (73.3)  27 (26.7) | 118 (84.9)  21 (15.1) | 0.01 |
| Ethical/moral objection to termination of pregnancy |  | | 87 (31.6) | 3 (3.0) | 16 (11.5) | <0.0001 |
| Referral for termination if objection |  | | 78 (88.6) | 3 (100.0) | 15 (93.8) | 0.69 |
| **Further management options** |  | |  |  |  |  |
| Palliative care |  | |  |  |  |  |
| Always/Sometimes  Only if asked/Never |  | | 244 (88.7)  31 (11.3) | 94 (93.1)  7 (6.9) | 126 (90.6)  13 (9.4) | 0.44 |
| Delivery in tertiary centre | |  | |  |  |  |
| Always/Sometimes  Only if asked/Never |  | | 171 (62.2)  104 (37.8) | 50 (49.5)  51 (50.5) | 81 (58.3)  58 (41.7) | 0.09 |
| Referral to paediatricians | |  | |  |  |  |
| Always/Sometimes  Only if asked/Never |  | | 256 (93.1)  19 (6.9) | 96 (95.1)  5 (4.9) | 131 (94.2)  8 (5.8) | 0.76 |
| Clinical ethics consultation | |  | |  |  |  |
| Always/Sometimes  Only if asked/Never |  | | 152 (55.3)  123 (44.7) | 28 (27.7)  73 (72.3) | 68 (48.9)  71 (51.1) | <0.0001 |
| Pastoral care | |  | |  |  |  |
| Always/Sometimes  Only if asked/Never |  | | 156 (56.7)  119 (43.3) | 47 (46.5)  54 (53.5) | 76 (54.7)  63 (45.3) | 0.21 |
| Fetal monitoring during labour | |  | |  |  |  |
| Always/Sometimes  Only if asked/Never |  | | 127 (46.2)  148 (53.8) | 38 (37.6)  63 (62.4) | 62 (44.6)  77 (55.4) | 0.33 |
| CS for fetal distress | |  | |  |  |  |
| Always/Sometimes  Only if asked/Never |  | | 99 (36.0)  176 (64.0) | 34 (33.7)  67 (66.3) | 50 (36.0)  89 (64.0) | 0.91 |
| Comply request for maternal-focus Obstetric care | | 271 (98.6) | | 100 (99.0) | 138 (99.3) | 0.79 |
| Counsel the patient if comply her request | |  | |  |  |  |
| Encourage  Support, not encourage  Neither encourage nor support  Discourage |  | | 58 (21.4)  130 (48.0)  77 (28.4)  6 (2.2) | 37 (37.0)  43 (43.0)  20 (20.0)  0 | 41 (29.7)  62 (44.9)  32 (23.2)  3 (2.2) | 0.053 |
| Comply request for fetal-orientated Obstetric care | | 218 (79.3) | | 77 (76.2) | 111 (79.9) | 0.77 |
| Counsel the patient if comply her request | |  | |  |  |  |
| Encourage  Support, not encourage  Neither encourage nor support  Discourage |  | | 6 (2.7)  87 (39.7)  54 (24.7)  72 (32.9) | 0  22 (28.6)  18 (23.4)  37 (48.0) | 2 (1.9)  37 (33.3)  31 (27.9)  41 (36.9) | 0.23 |
| Paediatrician present at birth | |  | |  |  |  |
| Routinely  If requested  Never |  | | 196 (71.3)  76 (27.6)  3 (1.1) | 64 (63.4)  33 (32.6)  4 (4.0) | 100 (71.9)  38 (27.4)  1 (0.7) | 0.22 |

*chi-squared test or Fisher’s exact test where appropriate

TABLE S3

Comparison of gender and duration of practice distribution of respondents with gender and age distribution in the broader population of consultant obstetricians in A/NZ and UK

| **Characteristics** | **Survey response** | | **Obstetricians** | |
| --- | --- | --- | --- | --- |
| Australia |  | % |  | % |
|  | Proportion male | 58 |  | 62^+^ |
|  | **(duration of practice as consultant)** |  | **(age group)** | % |
|  | <5 years  5-15 years  15-25 years  >25 years | 14.6  34.2  26  25 | 35-40  40-50  50-60  >60 | 14.3%  30.2  31.8  22.7 |
| UK |  |  |  |  |
|  | Proportion male | 40 |  | 49[^26^](#_ENREF_26) |
|  | **(duration of practice as consultant)** |  | **(age group)** | % |
|  |  |  |  |  |
|  | <5 years  5-25 years  >25 years | 16*  64  10 | 31-37  38-60  >60 | 16  74  10 |

+ personal correspondence, K. Lording 17/8/2012

We have assumed an average age of commencement of practice as a consultant obstetrician of 35 years for comparing duration of practice with age group. Age group categories are reported as available from UK and Australian/NZ primary sources.

*An additional 10% of UK respondents were still in training.

Age group data from

Australia: The specialist Australian and Gynaecology workforces in Australia 2004, ^48^

UK: Royal College of Obstetrics and Gynaecology survey 2010[^9^](#_ENREF_26)
